# Molecular identification and genotyping of *Blastocystis* in farmed Cattle, Goats, and Pigs from Zhejiang Province, China

**DOI:** 10.1016/j.fawpar.2025.e00280

**Published:** 2025-08-06

**Authors:** Wei Zhao, Lijie Sun, Yanbin Sun, Xinyi Fu, Shiyang Ma, Jiayin Zhang, Baolong Yan

**Affiliations:** a School of Basic Medical Sciences, Wenzhou Medical University, Wenzhou 325035, China; b Department of Clinical Laboratory, The Fifth Affiliated Hospital Sun Yat-sen University, Zhuhai 519000, China; c School of Public Health and Management, Wenzhou Medical University, Wenzhou 325035, China

**Keywords:** *Blastocystis*, Cattle, Goat, Pig, Genotypic variation, China

## Abstract

*Blastocystis* is a genus of parasitic protozoa that parasitize/colonize humans and animals gastrointestinal tract. The current study performed a molecular survey of *Blastocystis* in farm cattle (*Bos tarurs*), goats (*Capra hircus*), and pigs (*Susscrofa domestica*) raised in different cities in Zhejiang Province of China to better understand the epidemiology of *Blastocystis* in the animals of this region. A total of 859 fresh fecal samples were collected from 265 cattle, 386 goats, and 208 pigs. All these samples were screened for the detection of *Blastocystis* by amplifying the small subunit ribosomal RNA (*SSU rRNA*) gene *via* PCR and Sanger sequencing. Of the 859 samples tested, 12.1 % (104) were positive for *Blastocystis*, with cattle showing a prevalence of 19.6 % (52/265), goats with 11.4 % (44/386), and pigs with 3.8 % (8/208). Eight different subtypes of *Blastocystis* were found: ST10 (*n* = 51), ST5 (*n* = 11), ST12 (*n* = 9), ST14 (*n* = 8), ST21 (*n* = 8), ST23 (*n* = 7), ST26 (*n* = 6), and ST4 (*n* = 4). Cattle carried seven (ST5, ST10, ST12, ST14, ST21, ST23, and ST26), whereas goats harbored eight (ST4, ST5, ST10, ST12, ST14, ST21, ST23, and ST26) subtypes. All pig-derived *Blastocystis* isolates belonged only to ST5. These results are significant as they indicate that cattle, goats, and pigs in Zhejiang Province, China, harbor various subtypes of *Blastocystis*, which enhances our understanding of the distribution of *Blastocystis* among these hosts in China.

## 1 Introduction

Globally, *Blastocystis sp.* is a common unicellular eukaryote that is found in the intestines of humans and animals. Its taxonomic status and classification as a yeast, fungus, or cyst stage of another organism has changed over time. Most recently, it was reclassified as a protozoan belonging to the *Blastocystis* genus within the Stramenopiles clade (Aykur et al., 2024). The presence of *Blastocystis* can be associated with clinical symptoms (diarrhea, watery or loose stools, abdominal cramping, itching in the anus, weight loss, constipation, and excessive gas formation), but the majority of cases are asymptomatic (Kumarasamy et al., 2018). Nonetheless, the prevailing consensus is that *Blastocystis* is not a pathogen but rather a gut inhabitant that may even be beneficial (Deng and Tan, 2025a, Deng and Tan, 2025b). *Blastocystis* exhibits a robust association with healthy dietary patterns and enhanced cardiometabolic health, demonstrating links to improved cardiometabolic markers and suggesting potential protective effects against intestinal inflammation (Piperni et al., 2024). This contradiction in pathogenicity may stem from the extensive genetic diversity of *Blastocystis*, with different lineages of *Blastocystis* exhibiting varied ecological roles in the host gut (Deng et al., 2023; Lind et al., 2025). Studies have shown that ST4 is a beneficial commensal, and ST4 colonization can provide protection from colitis (Deng et al., 2022). In contrast, the eradication of *Blastocystis* subtype 3 has been found to improve the course of chronic spontaneous urticaria (Tuzer et al., 2025). Therefore, studying the subtype distribution of *Blastocystis* in various hosts is of paramount importance for gaining an in-depth understanding of its genetic variation and functional pathogenicity.

Presently, *Blastocystis sp.* is generally subtyped by examining polymorphic regions of its small subunit ribosomal RNA (*SSU rRNA*) gene (Stensvold and Clark, 2020). There are at least 44 subtypes of *Blastocystis* identified in humans and animals known globally (Santin et al., 2024). The subtypes that have been identified in humans include ST1 to ST10, ST12 to ST14, ST16, ST23, ST35, and ST41, all of these subtypes have also been identified in animals (Popruk et al., 2021; Hublin et al., 2021). Further, these subtypes show varying patterns of prevalence, for example, ST1 and ST2 is more common in non-human primates (Sanggari et al., 2022), ST4 in rodents and cats (Barati et al., 2022; Shams et al., 2022a), ST5 in pigs (Asghari et al., 2021), ST10 in cattle and goats (Shams et al., 2022b; Shams et al., 2021), and ST7 and ST6 in birds (Sanggari et al., 2022). This evidence indicates that various subtypes can coexist within different hosts, particularly showing an overlapping phenomenon between humans and animals. At the same time, there are also subtypes that are exclusive to specific hosts. Thus, understanding the distribution of *Blastocystis* subtypes across various hosts provides us with deeper insights into this organism.

Cattle, goats, and pigs are examples of farm animals that typically live in close proximity to humans and have been reported to carry multiple subtypes of *Blastocystis* (Asghari et al., 2021; Shams et al., 2022a; Shams et al., 2021). Most recently, cattle were known to harbor 16 different subtypes (ST1-ST7, ST10, ST12, ST14, ST17, ST21, and ST23-ST26) (Shams et al., 2022b). Similarly, it has been discovered that goats are host to ST1, ST3-ST7, ST10, ST12, ST14, ST21, ST23-ST26, and ST32 of *Blastocystis* (Shams et al., 2021). While domestic pigs were found to harbor nine genetically different *Blastocystis* subtypes (ST1-ST7, ST10, and ST15) (Asghari et al., 2021). Among the subtypes identified in these animals, some of them are zoonotic. This finding underscores the significant reservoir potential of cattle, goats, and pigs for a wide variety of *Blastocystis* subtypes, especially those that have zoonotic significance (Popruk et al., 2021; Asghari et al., 2021; Shams et al., 2022a; Shams et al., 2021). Therefore, to properly evaluate the impacts of *Blastocystis* infection on animals and effectively prevent its spread within the population, it is crucial to consistently monitor these animals.

China is a major contributor to animal husbandry and has an extensive industry for raising cattle, goats and pigs that covers the entire country. These animals inhabit rural areas, frequently living near humans, resulting in many chances for *Blastocystis* to spread between animals and humans. *Blastocystis* has been detected in cattle, goats, and pigs found in ∼12 provinces, mainly focused in the northern, western, and central regions of China (Table 1). Despite its widespread prevalence, a comprehensive understanding of the subtype diversity of *Blastocystis* in China continues to be a challenge, especially in regions such as Zhejiang Province where only one study based on the presence of *Blastocystis* in pig fecal samples, and no documented reports of *Blastocystis* in cattle and goats (Zou et al., 2021). Thus, this study aimed to screen the presence and subtype distribution of *Blastocystis* in cattle, goats and pigs in Zhejiang Province, China.

**Table 1:** The infection rate and subtype distribution of *Blastocystis* in pigs, goats and cattle from different provinces of China.

Host/Locations	No. positive/No. sampled (%)	Subtypes (no.)	References
Cattle			
Hebei	346/2746 (12.6)	ST1(37), ST2(1), ST5 (41), ST10 (159), ST14 (90), ST21 (14), ST26 (3)	Sun et al., 2024
Heilongjiang	54/526 (10.3)	ST4 (2), ST5 (1), ST10 (41), ST14 (10)	Zhu et al., 2017
	14/147 (9.5)	ST3 (2), ST10 (10), ST14 (2)	Wang et al., 2018
	8/20 (40.0)	ST3 (1), ST10 (2), ST26 (2), unknown subtypes (3)	Chen et al., 2023
	104/1632 (6.4)	ST1 (2), ST3 (18), ST5 (2), ST10 (61), ST14 (6), ST21 (1), ST24 (3), ST25 (2), ST26 (7)	Duan et al., 2024
Jiangxi	305/556 (54.9)	ST1 (9), ST5 (74), ST10 (189), ST14 (33)	Li et al., 2022
Northeast	17/803 (2.11)	ST10 (5), ST21 (1), ST23 (1), ST25 (2), ST26 (5)	Wang et al., 2021a
Shanxi	103/795 (13.0)	ST1 (4), ST10 (63), ST14 (8), ST21 (4), ST26 (21), unknown subtypes (3)	Liang et al., 2023

Pig			
Fujian	59/135 (43.7)	ST1 (2), ST3 (9), ST5 (45), ST14 (3)	Wang et al., 2021b
Guangdong	40/72 (55.6)	ST1 (1), ST5 (39)	Zou et al., 2021
Heilongjiang	28/39 (71.8)	ST1 (1), ST5 (19), Unknown (8)	Chen et al., 2023
	6/68 (8.8)	ST5 (6)	Wang et al., 2018
Hunan	19/83 (22.9)	ST1 (1), ST3 (2), ST5 (16)	Wang et al., 2021a
Jiangxi	316/1036 (30.5)	ST1 (23), ST3 (36), ST5 (242), ST14 (15)	Wang et al., 2021b
Shanxi	51/362 (14.1)	ST1(4), ST5(47)	Wei et al., 2023
Shaanxi	419/560 (74.8)	ST1 (15), ST3 (6), ST5 (397), ST10 (1)	Song et al., 2017a
Xinjiang	174/801 (21.7)	ST1 (7), ST3 (2), ST5 (165)	Wang et al., 2020
Yunnan	100/200 (50.0)	ST5 (100)	Zou et al., 2021
	433/866 (50.0)	ST1 (32), ST3 (19), ST5 (382).	Han et al., 2020
Zhejiang	30/124 (24.2)	ST5 (30)	Zou et al., 2021

Goat			
Guangdong	76/226 (33.6)	ST10 (50), ST14 (14), ST21 (6)	Yu et al., 2023
Heilongjiang	7/14 (50.0)	ST5 (6), unknown (1)	Chen et al., 2023
Inner Mongolia	72/692 (10.4)	ST5 (8), ST10 (37), ST14 (9), ST21 (2), ST26 (1)	Zhang et al., 2023
Shaanxi	458/789 (58.0)	ST1 (1), ST3 (1), ST4 (9), ST5 (31), ST10 (292), ST14 (123), unknown (1)	Song et al., 2017b
Tibet	22/260 (8.5)	ST1 (1), ST5 (2), ST6 (3), ST10 (16)	Chang et al., 2021

## 2 Materials and methods

### 2.1 Ethical approval

The protocol of this study was approved by the Research Ethics Committee and the Animal Ethical Committee of Wenzhou Medical University after a comprehensive review (SCILLSC-2021-01). All fecal sample was obtained exclusively with the consent of the owners or managers of the animals, assuring that no animals were harmed throughout the procedure.

### 2.2 Collection of fecal specimens

**Fig. 1:**
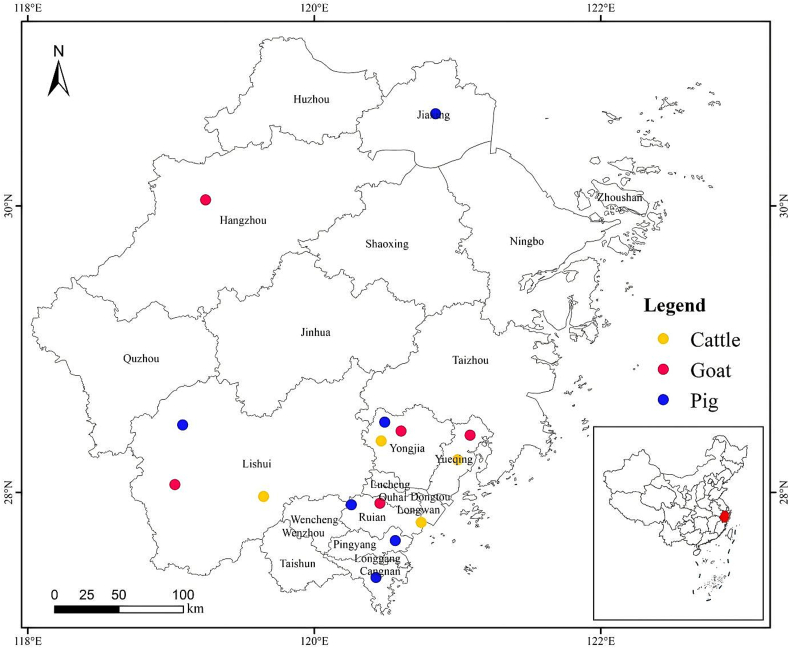
A map of the sampling locations in Zhejiang Province, China. This map was initially conceptualized and designed by the authors using ArcGIS 10.4 software. The original vector diagram imported into ArcGIS originated from the National Geomatics Center of China (http://www.ngcc.cn). The final version of the map, however, underwent modifications and assembly to meet specific attribution and permission guidelines. This was achieved using a combination of Microsoft PowerPoint 2003 and Adobe Photoshop CS6.

A total of 859 fresh fecal samples were collected between September 2021 and May 2023 from farms in Zhejiang Province, China. The study included the following animals: 265 cattle, 386 goats, and 208 pigs, all of which were raised intensively.The locations of these farms for cattle, goats and pigs were in four, five, and six communities, respectively (Table 2 and Fig. 1). The farms were selected primarily based on the owners' consent to participate and the convenience of reaching the animals for samples. All farms contained a single species of animals. All fecal sample was immediately collected from the ground after defecation, ensuring that any parts touching the ground were discarded to minimize the risk of cross-contamination with the ground or other animals. This process was carried out using sterile disposable latex gloves, and the samples were then deposited into individually labeled sterile tubes. The tubes were transported to the laboratory, assuring their preservation in a cold container with ice, and kept at 4 °C until they were prepared for processing.

**Table 2:** The infection rate and subtype distribution of *Blastocystis* in pigs, goats and cattle at different locations in Zhejiang Province, China.

Hosts	Location	No. Positive/No. sample (%, 95 % Confidence Intervals)	Subtype (n)	*P*-value
Pig	Cangnan	0/32 (0, 0–10.7)	/	⁎*P* = 0.45
	Jiaxing	2/36 (5.6, 1.5–18.2)	ST5 (2)	
	Lishui	3/37 (8.1, 2.8–21.3)	ST5 (3)	
	Pingyang	0/30 (0, 0–11.4)	/	
	Ruian	1/31 (3.2, 0.6–16.2)	ST5 (1)	
	Yongjia	2/42 (4.8, 1.3–15.8)	ST5 (2)	
	Subtotal	8/208 (3.8, 2.0–7.4)	ST5 (8)	
Goat	Hangzhou	18/92 (19.6, 12.8–28.8)	ST10 (12), ST23 (5), ST26 (1)	*P* = 0.03
	Lishui	4/88 (4.5, 1.8–11.1)	ST4 (3), ST26 (1)	
	Ruian	6/69 (8.7, 4.1–17.7)	ST21 (3), ST5 (1), ST10 (1), ST26 (1)	
	Yongjia	8/63 (12.7, 6.6–23.1)	ST10 (3), ST12 (1), ST14 (2), ST21 (1), ST26 (1)	
	Yueqing	8/74 (10.8, 5.6–19.9)	ST10 (4), ST4 (1), ST21 (2), ST26 (1)	
	Subtotal	44/386 (11.4, 8.6–15.0)	ST10 (20), ST21 (6), ST23 (5), ST26 (5), ST4 (4), ST14 (2), ST5 (1), ST12 (1)	
Cattle	Lishui	19/56 (33.9, 22.9–47.0)	ST10 (15), ST14 (3), ST21 (1)	*P* < 0.05
	Ruian	5/71 (7.0, 3.0–15.5)	ST12 (5)	
	Yongjia	12/71 (16.9, 9.9–27.3)	ST10 (9), ST5 (2), ST26 (1)	
	Yueqing	16/67 (23.9, 15.3–35.3)	ST10 (7), ST12 (3), ST14 (3), ST23 (2), ST21 (1)	
	Subtotal	52/265 (19.6, 15.3–24.8)	ST10 (31), ST12 (8), ST14 (6), ST5 (2), ST21 (2), ST23 (2), ST26 (1)	
Total		104/859 (12.1, 10.1–14.5)	ST10 (51), ST5 (11), ST12 (9), ST14 (8), ST21 (8), ST23 (7), ST26 (6), ST4 (4)	a*P* < 0.05

Note: The subtypes that are bolded in this text indicate the potential for zoonotic transmission.

⁎ Fisher's Exact Test.

a *P =* Pig *VS* Cattle *VS* Goat.

### 2.3 DNA extraction

We employed distilled water for diluting the feces, followed by the immediate filtering of the homogenates through a sieve with an 8.0-cm diameter and a pore size of 45 μm. The resultant filtrates were concentrated for 10 min at 1500 *g via* centrifugation. The genomic DNA of each processed fecal specimen was extracted directly from 200 mg using the QIAamp DNA stool mini reagent (QIAgen, Hilden, Germany). Based on the guidelines provided by the manufacturer, the extraction was carried out at a high lysate temperature of 95 °C to optimize the DNA yield (Zhao et al., 2014). The isolated DNA was preserved at −20 °C until further PCR analysis.

### 2.4 PCR amplification

All DNA samples were screened for *Blastocystis* detection through the amplification of a 500 bp nucleotide fragment, referred to as the Santín region, from the *SSU rRNA* gene of *Blastocystis*. The cycling parameters and primers employed in this procedure were the same as previously described (Santín et al., 2011). Forward primer BhRD1: 5’-GGAGGTAGTGACAATAAATC-3′ and reverse primer BhRD2: 5’-TGCTTTCGCACTTGTTCATC-3′. The cycling protocol involved a series of 35 cycles, with each cycle consisting of 95 °C for 30 s, 54 °C for 30 s, and 72 °C for 30 s. Further, a pre-heat phase conducted at 95 °C for 4 min was integrated, followed by a final extension phase lasting 5 min at 72 °C. For each PCR amplification, TaKaRa Taq DNA Polymerase (TaKaRa Bio Inc., Tokyo, Japan) was used. In all PCR assays, a negative control without any additional DNA was amplified to detect low-level contamination. Electrophoresis was performed on each of the PCR products using a 1.5 % agarose gel.

### 2.5 Nucleotide sequencing and analyzing

The PCR products that tested positive for *Blastocystis* were directly sequenced. The sequencing was carried out after purification on an ABI PRISM 3730 XL DNA Analyzer by Sangon Biotech Co., Ltd. (Shanghai, China), *via* the Big Dye Terminator v3.1 Cycle Sequencing Kit (Applied Biosystems, USA). Extra PCR products were sequenced, if required, for certain DNA preparations to validate the accuracy of the sequences *via* two-directional sequencing. Basic Local Alignment Search Tool (BLAST) and Clustal X 1.83 (http://www.clustal.org/) were used to align the nucleotide sequences obtained in the current study with reference sequences from the National Center for Biotechnology Information (NCBI) (https://www.ncbi.nlm.nih.gov/) to identify the subtype of *Blastocystis.*

### 2.6 Phylogenetic analysis

Phylogenetic analysis was performed to validate the group designation and compare the genetic association of subtypes of identified *Blastocystis* with the existing ones. This involved constructing a Neighbor-Joining (NJ) tree *via* the Mega X (http://www.megasoftware. net/) and measuring evolutionary distances using the Kimura-2-parameter model (Kumar et al., 2018). The stability of the tree was evaluated using bootstrap analysis with 1000 replicates.

### 2.7 Statistical analyses

Data was statistically examined using SPSS version 22.0 (SPSS, Chicago, IL, USA). A chi-square test or Fisher's exact test and 95 %confidence intervals (CIs) were employed to analyze and compare the prevalence of *Blastocystis* across various host species and farm groups. Statistical significance was determined for differences with *P*-values ≤0.05.

### 2.8 Nucleotide sequence accession numbers

Representative nucleotide sequences obtained in the present study were deposited in the GenBank database under accession numbers PP572669 to PP572697.

## 3 Results

### 3.1 Positivity rates of Blastocystis

Based on PCR and sequencing of the partial *SSU rRNA* gene, 104 of the 859 (12.1 %, 95 % CI: 10.1–14.5 %) fecal samples were positive for *Blastocystis*. The incidence of *Blastocystis* positivity was considerably increased in cattle (19.6 %, 95 % CI: 15.3–24.8 %, 52/265) than in goats (11.4 %, 95 % CI: 8.6–15.0 %, 44/386) and pigs (3.8 %, 95 % CI: 2.0–7.4 %, 8/208). These differences were statistically significant among the three species of animals (χ^2^ = 27.59, *P* < 0.05) (Table 2).

Among cattle, the highest infection rates were reported in Lishui (33.9 %, 95 % CI: 22.9–47.0 %, 19/56), followed by Yueqing (13.9 %, 95 % CI: 15.3–35.3 %, 16/67), Yongjia (16.9 %, 95 % CI: 9.9–27.3 %, 12/71) and Ruian (7.0 %, 95 % CI: 3.0–15.5 %, 5/71). There were statistically significant differences in infection rates among the four cattle farms examined (χ^2^ = 15.5, *P* < 0.05) (Table 2).

For goats, the highest infection rates were observed in Hangzhou (19.6 %, 95 % CI: 12.8–28.8 %, 18/92), followed by Yongjia (12.7 %, 95 % CI: 6.6–23.1 %, 8/63), Yueqing (10.8 %, 95 % CI: 5.6–19.9 %, 8/74), Ruian (8.7 %, 95 % CI: 4.1–17.7 %, 6/69) and Lishui (4.5 %, 95 % CI: 1.8–11.1 %, 4/88). The infection rates of the five goat farms under investigation varied significantly (χ^2^ = 10.8, *P* < 0.01) (Table 2).

Among pigs, Lishui had the highest infection rate (8.1 %, 95 % CI: 2.8–21.3 %, 3/37), followed by Jiaxing (5.6 %, 95 % CI: 1.5–18.2 %, 2/36), Yongjia (4.8 %, 95 % CI: 1.3–15.8 %, 2/42) and Ruian (3.2 %, 95 % CI: 0.6–16.2 %, 1/31), while, two farms from Cangnan and Pingyang did not report any *Blastocystis* infections. The Fisher's Exact Test conducted on the six pig farms did not reveal any statistically significant disparities in infection rates (*P* = 0.45) (Table 2).

### 3.2 Subtypes of *Blastocystis* isolates

A total of 104 *Blastocystis* positive-samples were analyzed using nucleotide sequence analysis of the partial sequence of the *SSU rRNA* gene (∼500 bp). These isolates were classified into eight subtypes: ST10 (*n* = 51), ST5 (*n* = 11), ST12 (*n* = 9), ST14 (*n* = 8), ST21 (*n* = 8), ST23 (*n* = 7), ST26 (*n* = 6), and ST4 (*n* = 4). Goats contained all the eight subtypes, specifically ST4, ST5, ST10, ST12, ST14, ST21, ST23, and ST26, while cattle harbored seven: ST5, ST10, ST12, ST14, ST21, ST23, and ST26. All *Blastocystis* isolates obtained from pigs have exclusively belonged to ST5 (Table 2).

In cattle, ST10 also holds a significant proportion, comprising 59.6 % (31/52) of samples. Next in frequency are ST12 (15.4 %, 8/52) and ST14 (11.5 %, 6/52). The frequency of the remaining subtypes is relatively low, occurring in a small number of instances in either 1.9 % (1/52) of ST26 samples or 3.5 % (2/52) of ST5, ST21, and ST23 samples, respectively. Importantly, there is variation in the prevalence of subtypes across cattle ranches. For instance, Yueqing farm harbors five subtypes (ST10, ST12, ST14, ST21 and ST23), Yongjia (ST5, ST10 and ST26) and Lishui (ST10, ST14 and ST21) both harbor three subtypes, while Rui'an has only one subtype (ST12) represented (Table 2).

In goats, the most prevalent subtype was ST10, comprising 45.5 % (20/44) of the isolates, followed by ST21 (13.6 %, 6/44), ST23 (11.4 %, 5/44), ST26 (11.4 %, 5/44), ST4 (9.1 %, 4/44), and ST14 (4.5 %, 2/44) in terms of frequency. Both ST5 and ST12 were detected in a single isolate (2.3 %, 1/44). Differences in the distribution of subtypes were noted among five goat farms. Specifically, ST26 was evenly spread across all farms, ST10 was found in four farms except for Lishui, ST21 was detected in three farms (Yongjia, Rui'an, and Yueqing), ST4 existed in Lishui and Yueqing, and ST23 was exclusively found in Hangzhou (Table 2).

Here all eight *Blastocystis* isolates obtained from pigs were solely classified as subtype ST5 (Table 2). Overall, goats and cattle showed the same subtypes, with ST10 being the most prevalent subtype in both species. Despite ST5 was also detected in cattle, goats, and pigs its occurrence was less frequent in the latter two species. Significantly, ST4 was the sole subtype observed only in goats in this study (Table 2).

### 3.3 Genetic diversity of *Blastocystis* subtypes

Among 104 recognized sequences, 29 representative sequences were observed. There were 10 sequences labeled as ST10 (ST10–1 to ST10–10), 10 sequences labeled as ST5 (ST5–1 to ST5–10), two sequences labeled as ST21 (ST21–1 and ST21–2), two sequences labeled as ST23 (ST23–1 and ST23–2), two sequences labeled as ST26 (ST26–1 and ST26–2), and one sequence each labeled as ST4, ST12, and ST14 (Table 3).

**Table 3:** Similarity analysis of ST sequences of *Blastocystis* obtained in this study.

SubST (n)	No. of samples containing variant	Accession number(s)	Similarity	Ref accession numbers in host from country
ST4 (4)	4	PP572669	100 %	OP725970 in human from Colombia
ST5 (11)	2	PP572670	100 %	MK244911 in cattle from the USA
	1	PP572671	100 %	ON834466 in bear from China
	1	PP572672	100 %	KF410604 in pig from the USA
	1	PP572673	100 %	OP725974 in human from Colombia
	1	PP572674	100 %	MK244910 in cattle from the USA
	1	PP572675	100 %	KF410597 in pig from the USA
	1	PP572676	99.58 %	MN472792 in *Struthio camelus* from Brazil
	1	PP572677	99.58 %	PP059244 in *Seland pony* from China
	1	PP572678	99.79 %	MH634445 in cattle from the USA
	1	PP572679	99.14 %	OR754922 in *Rattus tanezumi* from China
ST10 (51)	26	PP572680	100 %	MK244917 in cattle from the USA
	11	PP572681	100 %	MK244919 in cattle from the USA
	7	PP572682	100 %	MK244918 in cattle from the USA
	1	PP572683	99.38 %	MZ267647 in white-tailed deer from USA
	1	PP572684	99.58 %	MK244917 in cattle from the USA
	1	PP572685	97.35 %	KU981008 in *Cervus timorensis* from Malaysia
	1	PP572686	99.38 %	MN472782 in *Struthio camelus* from Brazil
	1	PP572687	96.66 %	OQ298897 in cattle from the USA
	1	PP572688	98.54 %	MK244919 in cattle from the USA
	1	PP572689	99.32 %	KU981009 in *Cervus timorensis* from Malaysia
ST12 (9)	9	PP572690	100 %	PP059262 in *Giraffa camelopardalis* from China
ST14 (8)	8	PP572691	99.37 %	MK244932 in cattle from the USA
ST21 (8)	7	PP572692	100 %	MK244935 in cattle from the USA
	1	PP572693	99.79 %	MK244935 in cattle from the USA
ST23 (7)	6	PP572694	100 %	MK244936 in cattle from the USA
	1	PP572695	98.75 %	MK244936 in cattle from the USA
ST26 (6)	5	PP572696	100 %	MK244947 in cattle from the USA
	1	PP572697	99.58 %	MK244947 in cattle from the USA

**Fig. 2:**
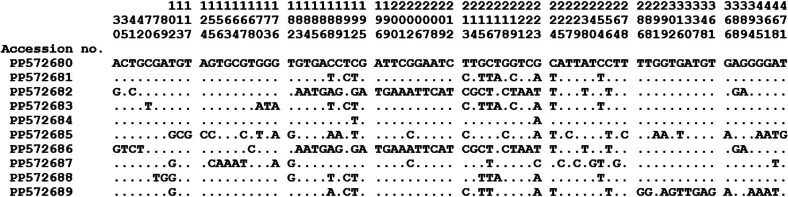
Sequence variation in the *SSU rRNA* gene among of *Blastocystis* ST10. The sequences of three known (GenBank: PP572680 to PP572682) and seven novel (GenBank: PP572683 to PP572689) identified in this study were aligned. The three rows of numbers preceding the nucleotide sequence correspond to the nucleotide at position.

Among the 10 ST10 sequences, three are reported: ST10–1 (PP572680, *n* = 26), ST10–2 (PP572681, *n* = 11) and ST10–3 (PP572682, *n* = 7) matches MK244917, MK244919 and MK244918, respectively All three reference sequences originate from Blastocystis ST10 isolated from cattle in the USA. The remaining seven representative sequences of ST10 (ST10–4 to ST10–10) are found in only one sample each. Novel genetic variants have been described in this study and differ from their known sequences by 96.66 % to 99.58 % (Table 3). Upon comparing the 10 representative sequences of ST10 obtained in this study, 78 nucleotide variations were discovered (Fig. 2).

Among the 10 representative sequences of ST5, a total of 17 polymorphic sites were found (Table 4). There have been six previously described sequences including, ST5–1 (PP572670) represents two samples and is completely identical to MK244911, which was identified in cattle from the USA. ST5–2 to ST5–6 (PP572671 to PP572675) each represent a single sample and have a 100 % similarity with ON834466 (found in bears from China), KF410604 (pigs from the USA), OP725974 (humans from Colombia), MK244910 (cattle from the USA), and KF410597 (pigs from the USA), respectively. The remaining four representative sequences, ST5–7 to ST5–10 (PP572676 to PP572679), were novel and differed from their respective known sequences by 1 to 6 bases (Table 3 and Table 4).

**Table 4:** Nucleotide variations at 17 polymorphic sites among the ten representative sequences of *Blastocystis* ST5 obtained in this study.

Accession no.	Nucleotide at position																
	52	197	206	207	208	217	349	423	439	441	442	448	449	451	461	463	470
PP572673	G	A	C	T	A	A	C	A	G	G	G	C	T	G	A	G	A
PP572678	G	A	C	T	A	G	C	A	G	G	G	C	T	G	A	G	A
PP572670	G	A	C	T	A	C	C	A	G	T	G	C	T	G	A	G	A
PP572675	G	A	C	T	A	G	C	A	G	T	G	C	T	G	A	G	A
PP572679	G	A	C	T	A	A	C	C	G	T	G	C	T	A	C	A	C
PP572672	G	G	A	A	T	A	C	A	G	T	G	C	T	G	A	G	A
PP572674	A	G	A	A	T	A	C	A	G	T	G	C	T	G	A	G	A
PP572676	G	G	A	A	T	A	C	A	G	G	G	T	T	G	A	G	A
PP572677	G	G	A	A	T	A	A	A	G	G	A	C	T	G	A	G	A
PP572671	G	G	T	A	A	A	C	A	A	T	G	C	C	C	A	G	A

The sequences of ST14 (PP572691), ST21 (PP572692 and PP572693), ST23 (PP572694 and PP572695), and ST26 (PP572696 and PP572697) demonstrate significant similarity to MK244932, MK244935, MK244936, and MK244947, respectively. All of these sequences originate from cattle in the USA. The similarities range from 98.75 % to 100 %. However, four samples of ST4 (PP572669) examined in this study demonstrate a strong correlation with OP725970, which originates from a human sample collected in Colombia. Furthermore, nine sequences of ST12 (PP572690) samples align precisely with PP059262, which is linked to *Giraffa camelopardalis* from China (Table 3).

**Fig. 3:**
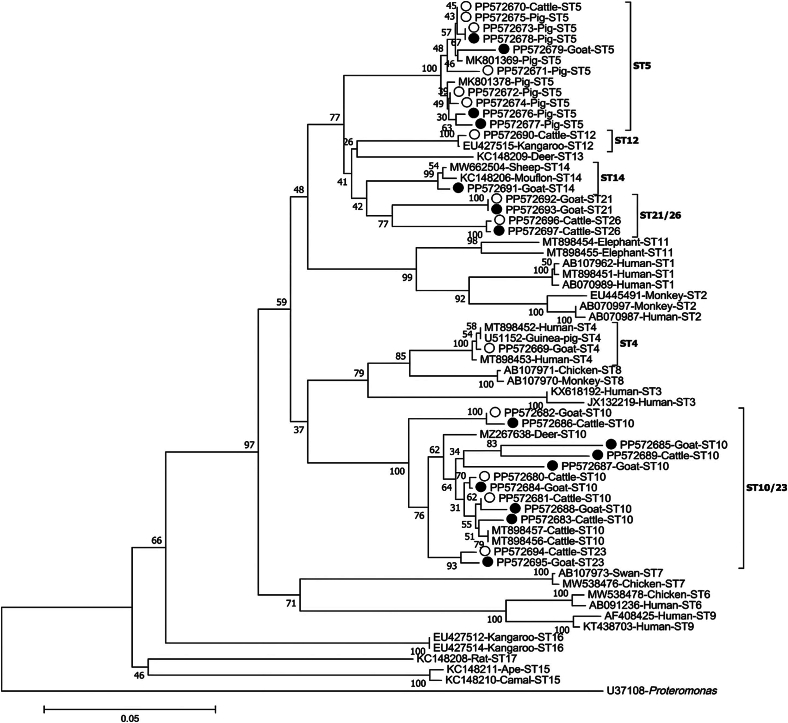
Phylogenetic analysis based on partial sequences of the *SSU rRNA* gene of *Blastocystis sp.*. In the tree, subtypes are denoted by hollow and solid circles, indicating known and novel sequences identified in this study, respectively. The tree was constructed using the Neighbor-Joining method, with the Kimura 2-parameter model serving as the foundation. To evaluate the reliability of the tree, bootstrap values were calculated from 1000 replicates.

Phylogenetic analysis, utilizing the NJ method, of nucleotide sequences isolated from *Blastocystis* subtypes in this study, demonstrated that the subtypes were distributed across distinct branches, with ST23 forming a cluster alongside ST10 (Fig. 3).

## 4 Discussion

The global prevalence of *Blastocystis* in cattle (24.4 %), goats (20.5 %), and domestic pigs (52.4 %) has been estimated, based on previous systematic reviews and meta-analyses (Asghari et al., 2021; Shams et al., 2022a; Shams et al., 2021). The study revealed that the infection rate in cattle was 19.6 %, in goats with *Blastocystis* it was 11.4 %, and among pigs, the rate was only 3.8 %. Overall, in comparison to infection rates documented in other research, the incident rates of infection in pigs and goats were comparatively reduced in the present study (Asghari et al., 2021; Shams et al., 2022a). Based on statistical analysis, infection with *Blastocystis* was significantly influenced by the species of host (Table 2). Infection rates for a specific host are influenced by many factors, including but not limited to the immune health of the animals, sample selection, seasonality, and examination techniques (Wang et al., 2018). The low infection rate in this study may be attributed to the following reasons: firstly, the farms investigated in this study are relatively hygienic, which provides a safer growth environment for animals and reduces the risk of infection; secondly, the animals selected in this study are all adults with relatively complete immune system and better resistance to pathogens; moreover, these animals were in a healthy state during sample collection, which further reduces the possibility of infection rate. It is noteworthy that each animal in this study was only collected once, which may have affected the detection rate to some extent, making the actual infection rate may be underestimated.

In the current study, eight different subtypes of *Blastocystis*: ST4, ST5, ST10, ST12, ST14, ST21, ST23 and ST26 were identified. Globally, ST10 is the subtype that is frequently observed in goats, sheep, and cattle (Shams et al., 2022a; Shams et al., 2021). Moreover, it is frequently observed in various companion animals, including cats, dogs, and horses, across the globe (Barati et al., 2022; Asghari et al., 2024). It has also been detected in other animals from China, including pigs, deer, bears, antelopes, chickens, swans, and wild birds (Hublin et al., 2021; Sanggari et al., 2022). Recently, there have been a few cases of human infections with ST10. These cases have been reported in Senegalese schoolchildren and Egyptians since 2020 (Khaled et al., 2020; Naguib et al., 2023). In a research conducted in Vietnam, it was found that ST10 is the second highest in humans, after ST3 (Nguyen et al., 2023). The implication is that ST10 has the capability to transmit across species, including humans.

Among the seven remaining subtypes, ST4, ST5, ST12, ST14, and ST23 have the ability to cause infection in both animals and humans. (Popruk et al., 2021; Hublin et al., 2021). Subtype 4 represents a prevalent subtype, especially approximately 19.8 % of human cases in Europe (Popruk et al., 2021). Moreover, diverse animal species, including mandrills, alpacas, artic foxes, bears, birds, bison, cats, cattle, deer, dogs, goats, New Zealand white rabbits, ring-tailed lemurs, pigs, have been identified as hosts of this subtype (Hublin et al., 2021; Sanggari et al., 2022; Rauff-Adedotun et al., 2021). Further, ST4 has been detected in various water sources in Asia (Rauff-Adedotun et al., 2021), and in China, ST4 has also been found in whooper swans, humans, rodents (including bamboo rats, porcupines, civets, and brown rats), and bears (Zhao et al., 2024). The identification of ST4 in four goats during the present study suggests the possibility of a transmission pattern involving goats, rodents, humans, and other animals. Almost all studies examining *Blastocystis* in pigs have identified ST5, which is one of the most prevalent subtypes (Asghari et al., 2021). Most significantly, this subtype is also capable of infecting humans (Popruk et al., 2021). Its zoonotic potential was demonstrated in China and Australia by the discovery of ST5 not only in pigs but also in their caretakers (Yan et al., 2007; Wang et al., 2014). It has also been documented that civets and other animals are susceptible to contracting this subtype (Zhao et al., 2023). This study marks the first instance of identifying the ST12 subtype in Chinese cattle and goats (Table 1). Previously, this subtype has been reported in humans across multiple countries, including China. Notably, the presence of ST12 has also been observed in other herbivores, such as yaks and sheep, within China. This discovery suggests that the ST12 subtype may be capable of transmitting between herbivores and humans. Given this potential for cross-species transmission, further investigation and exploration of this phenomenon are warranted. Occasionally reported in human cases ST14, and ST23 are prevalent genetic subtypes observed in cattle and sheep (Popruk et al., 2021; Hublin et al., 2021; Jinatham et al., 2021). They have been found in the environment, including in water and Soil in Thailand, although their host range remains unknown (Jinatham et al., 2021). The coexistence of these four subtypes in animals enhances the transmission of *Blastocystis* and potentially contributes to human infection. Further evidence of zoonotic transmission could be obtained by investigating the prevalence of their infection among the local population in the area under further examination.

Subtype 21 and ST26 are frequently observed in ruminants and were previously thought to be subtypes specific to ruminants (Asghari et al., 2021; Shams et al., 2022a). However, human infections have not been documented so far, and the extent of their zoonotic potential is unknown. Nevertheless, studies have shown that they can infect hosts other than cattle or goats, such as in *Elaphurus davidianus* and *Camelus bactrianus* from China, in White-Tailed Deer from the USA and in Eastern grey kangaroo and Emu from Australia (Ni et al., 2023; Yang et al., 2021; Koehler et al., 2023; Maloney et al., 2021). Their true host range, therefore, necessitates further study.

In this study, genetic diversity was observed within ST5 and ST10, whereas other ST subtypes showed relatively high conservation, characterized by high sequence identity among isolates of the same subtype. Out of the 11 ST5 subtypes, 10 distinct haplotypes were detected that differed by 17 variations throughout the genetic locus (Table 4). Similarly, 51 ST10 sequences were classified into 10 types, where 78 variants were found among them (Fig. 2). The emergence of these new sequences provides further evidence of the existence of extensive genetic variation even within the same subtype. Previous studies have demonstrated significant variation in sequences among ST10, resulting in the independent differentiation of many new subtypes (Santin et al., 2024). Hence, it is crucial for future research to fully investigate the sequencing of the complete small subunit genes of these unique sequences. This will allow for a more accurate classification, enabling a better understanding of their host range and route of transmission.

There are several limitations in this study. First, the selection criteria for farms were exclusively predicated on the owners' consent to participate and the convenience of accessing the animals for sampling, which may limit the generalizability of the findings to the conditions prevalent in Zhejiang Province of China. Second, it can be challenging to determine whether certain low-frequency subtypes are truly infection/colonization or simply carried by the animals due to the use of molecular methods without morphological evidence. Further, the infection rate can be underestimated because we only collected one fecal sample per animal, which may not have captured intermittent germ-cyst discharge. Last, the study did not include a population survey near these animals or environmental sampling around the farms, which means it did not provide direct evidence of *Blastocystis* contamination and its transmission to humans from one health standpoint. Despite there being some limitations, this study has successfully identified the existence of *Blastocystis* in cattle, goats and pigs, in the surveyed areas and offered initial observations regarding the subtype attributes of their genes.

## 5 Conclusions

Here is the inaugural study that delves into the prevalence and genetic signatures of *Blastocystis* in cattle, goats, and pigs hailing from Zhejiang Province in China. The discovery of zoonotic subtypes (ST4, ST5, ST10, ST12, ST14, and ST23) highlights the potential role played by cattle, pigs, and goats in the transmission of *Blastocystis* to humans. Despite the ongoing uncertainty surrounding the pathogenicity of *Blastocystis*, it is advisable to provide health education aimed at mitigating zoonotic risks to farmers, breeders, and veterinarians who have frequent contact with these livestock animals.

## CRediT authorship contribution statement

**Wei Zhao:** Writing – review & editing, Writing – original draft, Supervision, Software, Resources, Investigation, Funding acquisition, Formal analysis, Conceptualization. **Lijie Sun:** Writing – original draft, Software, Investigation, Formal analysis. **Yanbin Sun:** Writing – original draft, Investigation, Formal analysis. **Xinyi Fu:** Writing – review & editing, Investigation. **Shiyang Ma:** Writing – review & editing, Investigation. **Jiayin Zhang:** Writing – review & editing, Investigation. **Baolong Yan:** Writing – review & editing, Writing – original draft, Supervision, Resources, Formal analysis, Conceptualization.

## Funding

This work was supported by the Department of Education Scientific Research Project of Zhejiang (Y202249687), the Basic Scientific Research Project of Wenzhou (Y2023070). The funding sponsors had no role in study design, data collection and analysis, decision to publish, or preparation of the manuscript.

## Declaration of competing interest

The authors declare that they have no conflict of interest.

## Data Availability

The representative nucleotide sequences of *Blastocystis* obtained in the present study were deposited in GenBank database under the following accession nos.: PP572669 to PP572697.
